# Delirium Tremens

**Published:** 1874-05

**Authors:** 


*Delirium Tremens.*—The standard prescription for this condition is—

| | | |
|---|---|---|
| ℞.—Chloral hydrat, | . . . . | grs. xxx. |
| Potass. bromid, | . . . . | grs. lx. |
| M. | | |

